# A Low Molecular Weight Protein from the Sea Anemone *Anemonia viridis* with an Anti-Angiogenic Activity

**DOI:** 10.3390/md16040134

**Published:** 2018-04-19

**Authors:** Erwann P. Loret, José Luis, Christopher Nuccio, Claude Villard, Pascal Mansuelle, Régine Lebrun, Pierre Henri Villard

**Affiliations:** 1 Aix-Marseille University (AMU), Université d’Avignon, Centre National de la Recherche Scientifique (CNRS), Institut de la Recherche et du Développement (IRD), Institut Méditerranéen de Biologie et d’Ecologie. CNRS UMR 7263 IRD 237 Faculté de Pharmacie, 27 Bd Jean Moulin, 13385 Marseille, France; pierre.villard@univ-amu.fr; 2 AMU, CNRS, Institut de Neurophysio Pathologie, 13385 Marseille, France; jose.luis@univ-amu.fr (J.L.); claude.villard@univ-amu.fr (C.V.); 3 AMU, Institut National de la Santé Et de la Recherche Scientifique, 13385 Marseille, France; christopher.nuccio@univ-amu.fr; 4 AMU, CNRS Formation de Recherche 3479, Institut de Microbiologie de la Méditerranée, Plateforme Protéomique, 31 Chemin Joseph Aiguier, 13402 Marseille, France; pmansuelle@imm.cnrs.fr (P.M.); rlebrun@imm.cnrs.fr (R.L.)

**Keywords:** sea anemone, drug discovery, cancer, antiangiogenic, endothelial cells, RGD motif, kunitz type inhibitor

## Abstract

Sea anemones are a remarkable source of active principles due to a decentralized venom system. New blood vessel growth or angiogenesis is a very promising target against cancer, but the few available antiangiogenic compounds have limited efficacy. In this study, a protein fraction, purified from tentacles of *Anemonia viridis*, was able to limit endothelial cells proliferation and angiogenesis at low concentration (14 nM). Protein sequences were determined with Edman degradation and mass spectrometry in source decay and revealed homologies with Blood Depressing Substance (BDS) sea anemones. The presence of a two-turn alpha helix observed with circular dichroism and a trypsin activity inhibition suggested that the active principle could be a Kunitz-type inhibitor, which may interact with an integrin due to an Arginine Glycin Aspartate (RGD) motif. Molecular modeling showed that this RGD motif was well exposed to solvent. This active principle could improve antiangiogenic therapy from existing antiangiogenic compounds binding on the Vascular Endothelial Growth Factor (VEGF).

## 1. Introduction

Sea anemones have been understudied as a source of new pharmacological tools or therapeutic leads [1]. Sea anemones belong to *Cnidaria* that also includes corals, jellyfish and sponges. They have toxic peptides to incapacitate and immobilize prey and to defend from potential predators [2]. Their toxin arsenal is complex, targeting a variety of pharmacological targets such as ionic channels, inflammatory receptors [3] or pore forming protein in cellular membranes [4]. Anti-hyperglycemic and anti-diabetic activities were also observed from sea anemone extract [5]. 

Active principles from venomous species such as snakes or scorpions have a centralized venom system and have a structural homogeneity, which is not the case for sea anemones. Scorpion active principles for instance have a scaffold characterized by three beta sheets, an alpha helix and four disulfide bridges [6]. In sea anemones, the venom system is decentralized in all parts of the animal body showing a higher diversity in size and scaffold of protein active principles. The active principles binding on receptors such as ionic channels are short size proteins between 3000 and 5000 Da cross linked with three disulfide bridges [2]. They have different structural scaffolds regarding their pharmacological targets. Active principles binding on ionic channel are characterized by three beta strands [7], while those binding on enzymes such as the family of Kunitz-type inhibitors have two beta strands and an alpha helix [8]. Surprisingly, the sea anemone toxin Bg1, with a scaffold completely different, can compete at nanomolar concentrations with the Aah II scorpion toxin on the same pharmacological site on sodium channel [9]. However, the superposition of the 3D structure of these two proteins shows that the lateral chains of four basic residues are in exactly the same positions [9].

Cancer treatments are mainly based on anti-mitotic compounds that have side effects because they block the division of both cancer cells and healthy cells. Cancer cells need to be vascularized by endothelial cells, in a process called angiogenesis, to grow as a tumor and then to spread as metastases inducing the patient death. It was proposed almost 50 years ago to block angiogenesis to fight against cancer because angiogenesis is no longer important after embryogenesis [10]. There are very few antiangiogenic compounds compared to antimitotic compounds and it has only been a decade since antiangiogenic compounds began being tested in clinical trials. Amazingly, antiangiogenic compounds showed a limited efficacy because they all bind on the Vascular Endothelial Growth Factor (VEGF) or the VEGF receptor and tumor cells can trigger different biological ways to have angiogenesis [11]. Among antiangiogenic compounds binding on VEGF, the most used in clinical trial is Bevacizumab (known as Avastin), which is a monoclonal antibody [11]. Bevacizumab is costly due to its size and difficulties to have germ free production as recombinant protein. Furthermore, resistance to Bevacizumab is observed due to the upregulation of other redundant angiogenic factors different from VEGF [12]. Compounds binding on VEGF receptors that are not proteins have toxic effects that limit their use. Resistance towards VEGF-centered antiangiogenic therapy represents a substantial clinical challenge [10]. There is therefore a need to have compounds blocking angiogenesis that do not bind on VEGF or VEGF receptor, are easy to produce and have no long-term toxicity. 

Short size synthetic proteins (less than 50 residues) are suitable compounds for this goal. *Anemonia viridis* (also called *Anemonia sulcata*) is well studied and proven to be a remarkable source of low molecular proteins with different pharmacological binding sites on ionic channel receptors [7]. Seven short size proteins (42–49 residues) binding on ionic channels were purified and characterized from *Anemonia viridis* [7]. Among them, AS2 is a 47-residue-long protein (4934 Da) having only beta structures [13]. There is also BDS-1 that is a blood depressing protein of 43 residues (4708 Da) binding on a specific potassium channel [14]. The main secondary structure of BDS-1 is a triple-stranded antiparallel beta-sheet without alpha helix [7]. Recently, a partially purified extract of *Anemonia viridis* was reported to affect the growth and viability of selected tumor cell lines [15].

In this study, two cellular antiangiogenic screening tests were used with Human Microvascular Endothelial Cells (HMEC) having the Epithelial Growth Factor instead of VEGF [16]. The first one measured the proliferation of HMEC and the second one the formation of HMEC capillary network. These screening tests made it possible to identify from *Anemonia viridis* a protein fraction able to limit HMEC proliferation and angiogenesis at low concentration (IC_50_ 14 nM). Trypsin inhibition and the presence of a two turns alpha helix revealed by circular dichroism suggest that the active principle is a kunitz type inhibitor. 

## 2. Results

### 2.1. Purification from Full Sea Anemone Body

To preserve the integrity of potential protein active principles, a new purification protocol was established. This protocol described in the experimental procedure was tested with both the full animal body and tentacles in preliminary studies using two animal bodies. HPLC analysis after precipitation, centrifugation and filtrations steps revealed that tentacles had the highest content in short size proteins compared to the full body (data not shown). Tentacles were therefore chosen to obtain pure proteins with semi preparative HPLC. Before HPLC, it was necessary to carry out different steps of extractions and filtrations. To avoid protein enzyme digestions or degradations, the tentacles from 11 *Anemonia viridis* were cut and immediately frozen at −20 °C. After 24 h at −20 °C, the tentacles were lyophilized for three days. Then, the dry tentacles (20.8 g) were mashed*.* A first precipitation and a centrifugation were carried out with a hydrophobic solvent to eliminate lipids and membrane proteins. After evaporation for 24 h, the weight of the pellet was 19.2 g. The pellet was suspended in water 0.1% TFA and a centrifugation was carried out to eliminate the calcic skeleton. A gel filtration (cut off 30 KDa) and a 0.22 µm filtration made it possible to select low molecular weight compounds. A UV absorption spectrum of this solution (data not shown) revealed two major bands at 265 nm and 330 nm due to alkaloid pigments that are low molecular weight compounds abundant in sea anemones [17]. After lyophilization, only 0.984 g remained from the initial 20.8 g dry tentacles (4.7%). Semi preparative HPLC purification was carried out on this 0.984 g and made it possible to obtain 15 fractions. The first fraction contained essentially alkaloid pigments. The total amount of proteins found in the other fractions was only 30 mg. The low molecular weight proteins that are the targets of this purification corresponded to 0.14% of the dry tentacles in the beginning of this purification protocol.

**Figure 1:**
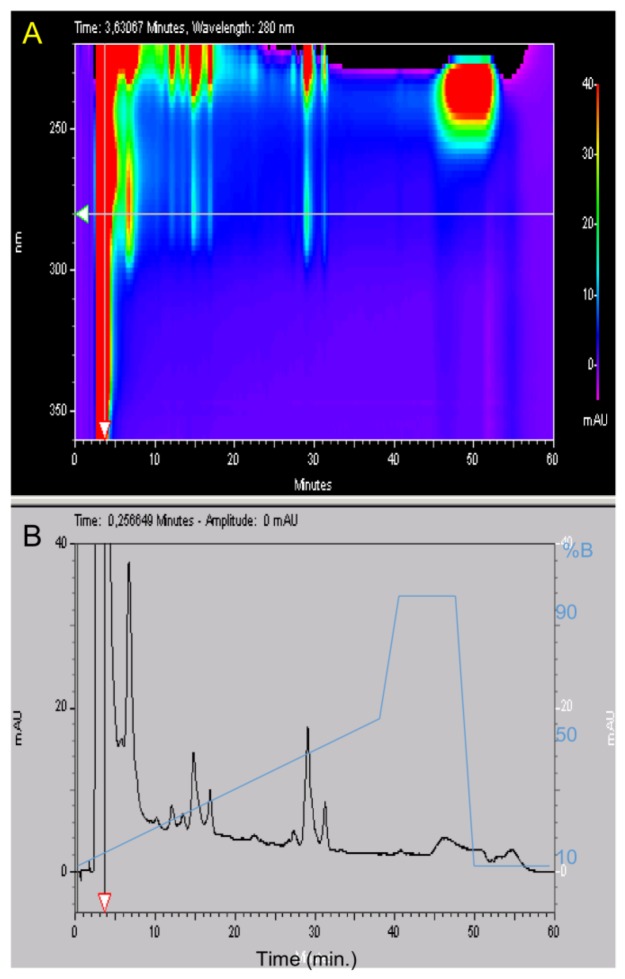
3D Analytical HPLC of *Anemonia viridis* tentacles after phase extractions, centrifugation and filtrations and before semi preparative HPLC. The UV absorbance of the effluent was measured with a diode ray detector. (**A**) A 3D plot displaying elution time from 0 to 60 min (*x* axis) regarding wavelengths from 220 to 330 nm (*y* axis). The *z* axis corresponds to different peak intensities and is represented with a color scale going from 0 (purple) to 40 mAU (red). The horizontal arrow shows the 280 nm wavelength. (**B**) Elution time regarding absorbance at 280 nm from 0 to 40 mAU and the acetonitrile (%B) gradient in blue. The vertical arrow shows the retention time of the major peak at 4 min that is out scaled with an absorbance at 500 mAU.

HPLC analysis before semi preparative HPLC purification showed a major peak at 4 min (Figure 1B) and the spectral analysis in (Figure 1A) revealed a major absorbance at 265 nm and 330 nm that were dominant in the spectral analysis carried out before semi preparative HPLC. This was not a protein spectrum. Protein spectra are characterized by a first absorbance at 280 nm due to aromatic residues and then a major absorbance due to peptidic bonds that begins at 210 nm [16]. The elution time showed that these compounds were not retained in C8 column and confirmed that they were alkaloid pigments with aromatic cycles [18].

Semi prepartive HPLC made it possible to collect nine main protein fractions. Mass spectroscopy analysis revealed that the molecular weight of these proteins was going from 4690 to 6700 Da. They displayed typical protein UV spectra (data not shown). The most abundant fraction eluted at 7 min (Figure 1) and had an absorbance spectrum with a bump at 290 nm typical of tryptophan (W) that were highly conserved in active principles binding on ionic channel identified in *Anemonia viridis* but the molecular weight of this protein (6620 Da) was not compatible with known *Anemonia viridis* active principles that had sizes <50 amino acid residues [6]. The other proteins eluting from 7 min to 15 min displayed absorbance spectra without W contribution characterized by a shift of the maximum absorbance from 280 to 278 nm (data not shown). Other fractions had W contribution, particularly the 31-min fraction. It was possible to recognize and isolate AS2 in this fraction due to its molecular weight and its N-terminal sequence, which was a 47-residue-long protein having three W in its sequence [12].

### 2.2. HMEC Proliferation Inhibition (n = 2)

**Figure 2:**
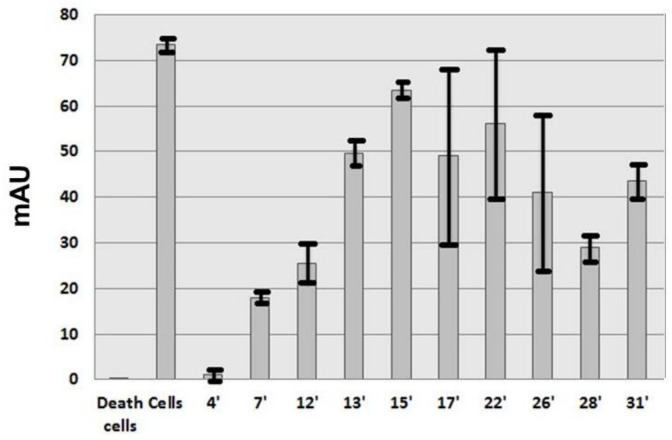
HMEC proliferation inhibition with 50 µg/L of the alkaloid fraction (4 min) and the nine protein fractions purified from semipreparative HPLC. This experiment was carried out twice with two different purifications. Alkaloid pigments were present in fraction 17 min, 22 min and 26 min in the second purification and may explain the differences between the two experiments.

A screening was carried out with the nine protein fractions and the alkaloid pigments as control to measure the capacity of these proteins to inhibit HMEC proliferation. This experiment was performed twice with fractions from the second purification. The test was carried out with a 10 µM concentration for the nine protein fractions and the alkaloid pigments. It was important in this test that living cells remained detectable to differentiate between cytotoxicity and proliferation inhibition. Proliferation inhibition was characterized by a decrease of absorbance (Figure 2). The absorbance was proportional to the mitochondrial activity. Only three protein fractions were able to limit HMEC proliferation up or below to 50% at 7 min, 12 min and 28 min (Figure 2). No absorbance due to cellular death was observed with the alkaloid pigments (Figure 2, 4 min). Cytotoxicity of *Anemonia viridis* alkaloid pigments was previously reported [17]. Trace of alkaloid pigments was observed in analytical HPLC of 7 min and 12 min proteins (data not shown) and the decrease of absorbance >50% could be due to cytotoxicity and not to an inhibition of proliferation due to an antiangiogenic activity. Alkaloid pigments were present in fraction 17 min, 22 min and 26 min in the second purification and may explain the high variation between the two experiments.

### 2.3. HMEC Tubulogenesis Assay (n = 3)

**Figure 3:**
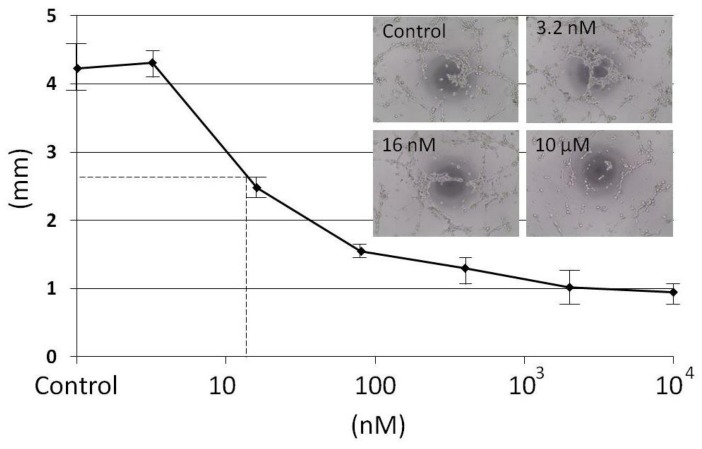
HMEC tubulogenesis assay with the 28-min fraction with concentrations going from 10 µM to 3.2 nM in a triplicate experiment (*n* = 2). A network with interconnected lines similar to control was observed only at 3.2 nM. The dose–response curve was established by measuring capillary length with the METAMORPH software. The six dilutions were represented with the control in a decimal logarithm scale regarding the capillary length measured in mm. The half effect corresponding to a capillary length of 2.65 mm was observed at 14 nM.

A first screening at 10 µM made with the fractions obtained from SP HPLC showed that the capillary network formation was inhibited only with the 28-min fraction and not with the 7 min- and 12-min fractions where the remaining living cells were able to display a network (data not shown). To measure a dose effect of the 28-min fraction on HMEC tubulogenesis, seven dilutions from 10 µM to 3.2 nM (dilution factor 1/5) were tested (Figure 3). A capillary network with interconnected lines similar to control is observed only at 3.2 nM (Figure S1 in Supplementary Materials). To determine the half effect, a dose–response curve was established by measuring the capillary length with METAMORPH 7.6 software for each dilution (Figure 3).

### 2.4. Primary Structure Analyses

The Edman N-terminal sequencing of the 28-min fraction made it possible to identify the first 31 amino acid residues and revealed a sequence homology with BDS sea anemones [7]. The sequence was identical to BDS-1 excepted on position 18 with a phenylalanine (F) instead of Leucine (L) in BDS-1. Although the 28-min fraction gave only one peak in analytical HPLC, the sequencing revealed the presence of contaminants with a proline (P) instead of S7 and glycine (G) instead of Lysine (K) 9. The mutation S/P 7 was observed in BDS2 [7].

**Figure 4:**
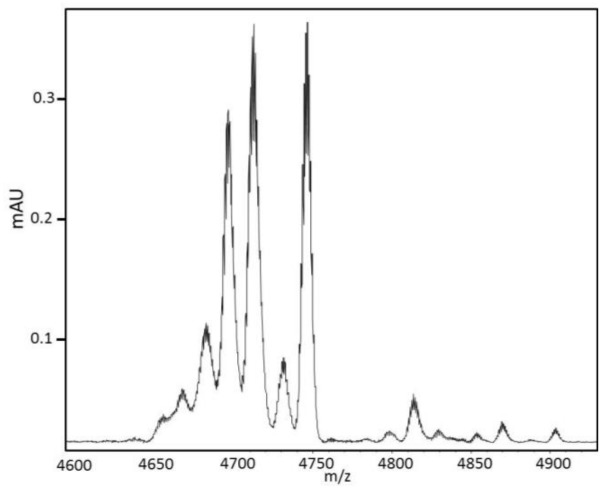
High resolution MALDI MS of the 28-min fraction. Each peak was decomposed in different isotopic forms. The isotopic average masses of the three main peaks were, respectively, 4692, 4708 and 4742 Da. Their mono isotopic masses were, respectively, 4689, 4705 and 4739 Da.

A first Matrix-Assisted Laser Desorption Ionization Mass Spectrometry (MALDI MS) at low resolution gave a molecular weight of 4730 Da for the 28-min fraction. However, high resolution MALDI MS revealed that the 28-min fraction was most heterogeneous and contained up to ten proteins (Figure 4). The three main proteins had average molecular weights corresponding to 4692, 4708 and 4742 Da. To further investigate on the monoisotopic mass of the protein content, the 28-min fraction was also analyzed with high resolution Orbitrap Mass Spectrometry (Figure S2 in Supplementary Materials).

It was possible to determine the sequences of these three main proteins in the 28-min fraction with both MALDI MS using In Source Decay (ISD) technology and HPLC-MS/MS analyses of the digested fractions after Trypsin treatment (Figures S3–S5 in Supplementary Materials). ISD confirmed the sequence homology between BDS1 and BDS5 from the N-terminal Alanine to F18 for BDS5 and L18 for BDS1. The experiment revealed a third chain (BDS16) with a F18 as BDS5 but with a difference of 50 Da compared to BDS5. A bottom up approach using HPLC-MS/MS was performed using a digest of the fraction (Figure S4). The major peaks validated ISD results and identified a peptide with minus 50 Da regarding the masses of the N-terminus of BDS5 and BDS1. This peptide corresponding to the N-terminus of BDS16 was sequenced with ISD (Figure S5) to provide the full sequence of BDS16. The three proteins identified in the 28-min fraction were therefore BDS-1 and two others proteins named BDS-5 and BDS-16.


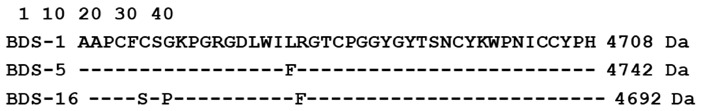


**Figure 5:**
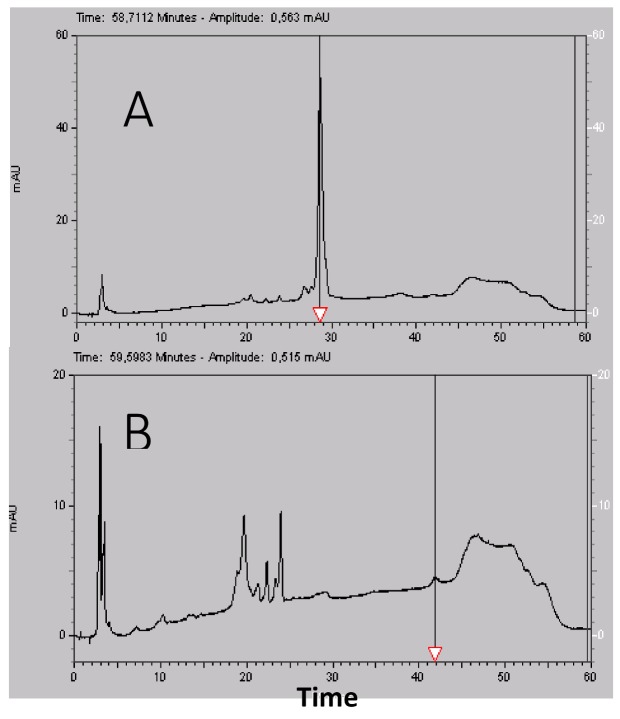
Enzymatic digestion of the 28-min fraction with a Trypsin/Lys C mixture at 37 °C for: one hour (**A**); and three days (**B**). The 28-min fraction displayed only one peak and remained almost unchanged after one-hour digestion, while it should have completely disappeared. This experiment suggested that the 28-min fraction contained a Trypsin inhibitor. The Trypsin/Lys C mixture eluted at 42 min. Another digestion experiment with only Trypsin gave the same results with 72 h necessary to have a complete digestion (Figure S6 in Supplementary Materials).

Enzymatic digestion was carried out on the 28-min fraction with a Trypsin/Lys C mixture. The disulfide bridges were not altered to allow the possibility of identifying their location. Amazingly, in regular condition corresponding to 1 h incubation at 37 °C, the HPLC peak of the 28-min fraction remained almost unaltered (Figure 5A). It was necessary to wait three days in similar conditions to observe the complete digestion of the 28-min fraction. Resistance to Trypsin digestion was not reported when BDS-1 was sequenced [14]. Sequencing of the peptides purified after complete digestion of the 28-min fraction revealed that the peaks between 22 min and 24 min were the same sequence **^13^GDLWIFR^19^** corresponding to the central part of BDS-5. The peptide corresponding to this sequence had probably different conformational states that may explain the different elution times observed between 22 min and 24 min. The Edman sequencing either for the N-terminus or in peptides obtained from enzymatic digestion did not confirm in the 28-min fraction, the presence of BDS-1 characterized by L18. Sequencing of the peak eluting at 20′ revealed the presence of three peptides corresponding to expected sequences, the C terminus with **^35^WPNICCYPH^43^**, the N-terminus (**^1^AAPCFCSGK^9^**) and **^20^GTCPGGYGYTSNCYK^34^**. The gap between **^31^NCYK^34^** was determined by ISD. It was not possible to identify a peptide corresponding to the **^10^PGR^12^** sequence but this part of the BDS-5 sequence was previously identified in the N terminal sequencing of the 28-min fraction. Another digestion of the non-reduced 28-min fraction was performed at different times to control from 20 h to 70 h (Figure S6 in Supplementary Materials). The three peptides produced four different peptides, two for the peptide 13–19 of BDS-5 and BDS-1 and two others with an average mass of 3842 and 3892 attesting that all the other peptides were crosslinked by disulfide bridges. It was possible to deduce that the three disulfide bridges of BDS-5 were similar to BDS-1 and corresponded to **^4^C**–**C^39^**, **^6^C**–**C^32^** and **^22^C**–**C^40^**.

### 2.5. Secondary and Tertiary Structure Analyses

**Figure 6:**
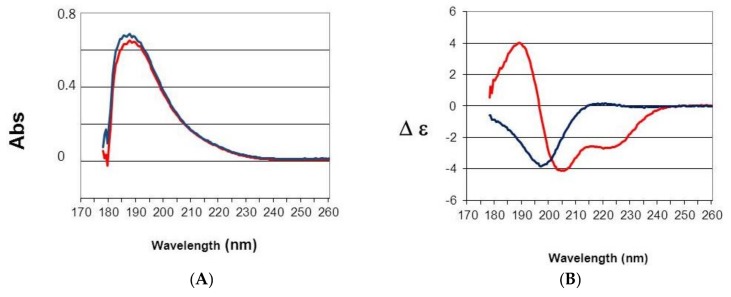
Far UV (178–260 nm) absorbance (**A**); and circular dichroism (**B**) spectra of AS2 (Blue) and the 28-min fraction (red). CD spectra are presented as ∆ε per amide rather than in ∆A unit to have a measure independent of mass and concentration [17].

**Figure 7:**
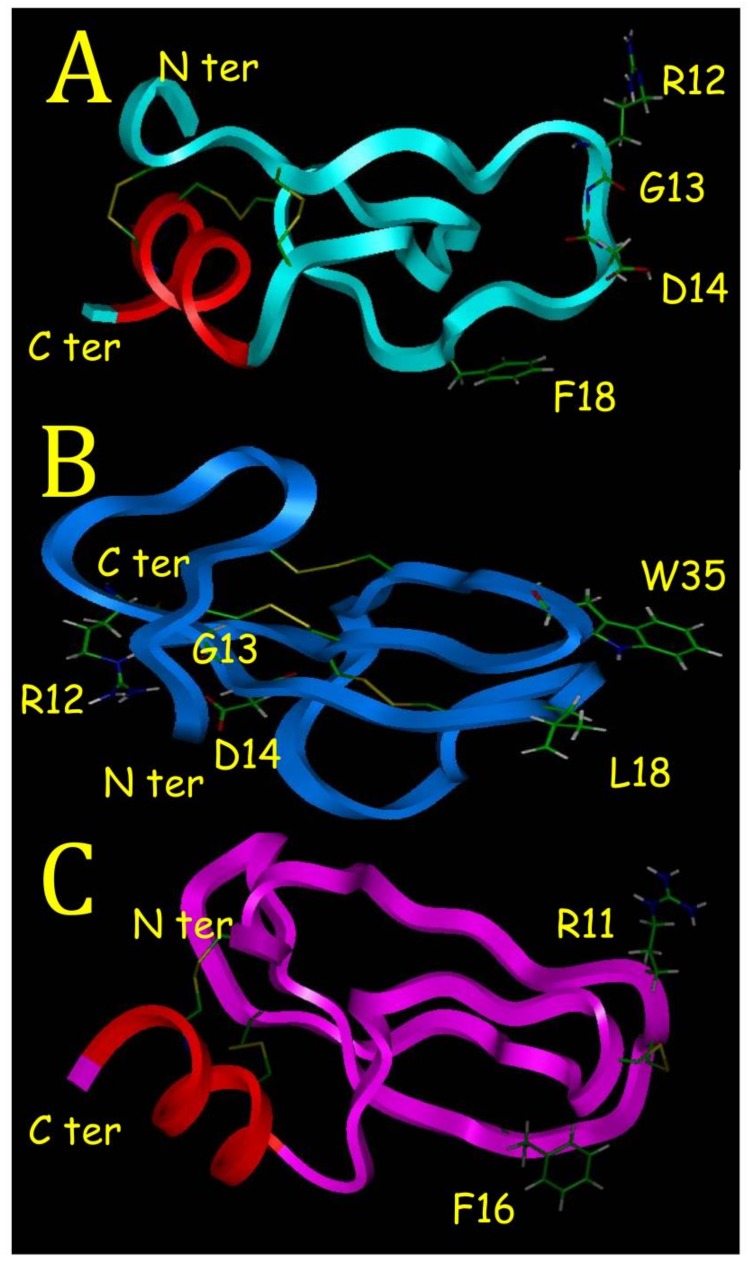
(**A**) The BDS-5 model 3D structure determined with molecular modeling from atomic coordinates of SHPI-1 [18] in (**C**). It was also possible to build a BDS-5 model from BDS-1 structure (**B**) since only one mutation F/L 18 existed between these two proteins but this model was not compatible with CD data. Although one disulfide bridge (colored in yellow) is different in SHPI-1 structure, molecular modeling showed that it is possible to have a BDS-5 model structure compatible with a Kunitz-type structure characterized by a C-terminal short alpha helix (colored in red).

A far UV study (178–260 nm) of AS2 and the 28-min fraction was carried out (Figure 6). Absorbance spectra were similar and characterized by absorption at 190 nm of the π–π* transition of the peptide bonds amid chromophore. A bump due to n–π* transition was observed at 210 nm [17]. The absorption spectra revealed that the protein concentrations were similar and the light transmission was correct due to the linearity of the signal up to 180 nm (Figure 6A). CD spectra revealed a major difference between AS2 and the 28-min fraction, thus a difference in the content in secondary structures (Figure 7B). AS2 had a CD spectra characteristic of beta sheet and beta turns with no alpha helix. This type of folding is typical of *Anemonia viridis* proteins binding on ionic channel such as AS2 or BDS-1 [7]. The shift of this negative band at 196 nM showed that this was a well-structured protein characterized by beta structures in accordance with a former Laser Raman structural study made with AS2 [13]. The CD spectrum of the 28-min fraction was characteristic of an alpha helix with the splitting of the π–π* transition in two bands, a positive one at 190 nm and a negative one at 205 nm, and a negative band at 215 nm due to n–π* transition [17]. The low intensity of the CD bands and a bump at 200 nm in the positive band revealed contributions of beta structures [17]. The CD spectrum of BDS-1 had no positive band and was typical of CD spectra with only beta structures [7]. The alpha helix CD signal was due probably to BDS-5 and might correspond to a two-turn alpha helix [17].

Based on CD data, the BDS-5 model was built from the crystal structure of SHPI-1 in a complex with Elastase [18]. SHPI-1 was purified from the *Stichodactyla helianthus* sea anemone [18]. This structure model was chosen because it was a low molecular weight protein (55 residues) cross linked with three disulfide bridges and the content in secondary structures was compatible with CD data, which was not the case for BDS-1 [7]. The atomic coordinates of alpha carbons of SHPI-1 N-terminus (residues 1–15) and C-terminus of SHPI-1 (residues 32–55) were used as template for BDS-5. Then, atomic coordinates of lateral chains of BDS-5 residues were assigned without overlap to avoid energy bumps. Ten loops were generated to connect BDS-5 residues 15–20. The lowest root means square deviation loop regarding optimum dihedral angles was used to assign atomic coordinates to the last five missing BDS-5 residues to complete the final structure.

A first energy minimization and dynamic procedure was carried out with only one disulfide bridge (**^4^C**-**C^39^**) to connect the N and C terminus of BDS-5 with the four other cysteines being free. Structural changes due to dynamic procedure and the constraint related to this **^4^C**-**C^39^** disulfide bridge made it possible to have the SH group at the right position to constitute two other disulfide bridges, **^6^C**-**C^32^** and **^22^C**-**C^40^**. A final energy minimization procedure made it possible to obtain a final BDS-5 model 3D structure with a low van der Waals energy (Figure 7). Kunitz-type structures are low molecular weight proteins characterized by a short alpha helix, a two-beta strand sheet and three disulfide bridges as the SHPI-1 sea anemone protein [18]. Molecular modeling showed that it was possible to have a BDS-5 model 3D structure compatible with a Kunitz-type structure with disulfide bridges similar to BDS-1. Attempt to create a BDS-5 model using a first disulfide bridge constraint with **^4^C**-**C^40^** was not possible because it induced the collapse of the C terminal alpha helix to constitute the two other disulfide bridges. It is important to note that another BDS-5 model was possible from BDS-1 NMR structure [7]. In that case, the BDS-5 model was similar to BDS-1 with the F18 lateral chain located at the L18 position (Figure 7B). In the two BDS-5 models, F18 appeared located similarly (data not shown). It is interesting to note that F16 in the SHPI-1 crystal structure had also a similar position (Figure 7A,C). R11 and F18 in SHPI-1 were the residues involved in the inhibition of Elastase [18], and the BDS-5 3D structure model showed that R12 was located similarly to R11 in SHPI-1 (Figure 7A,C). The presence of a C terminal alpha helix induced a major change in the location of the **^12^RGD^14^** motif for the BDS-5 structure model (Figure 7A) compared to BDS-1 NMR structure (Figure 7B). Contrary to BDS-1, the **^12^RGD^14^** motif in the BDS-5 structure model was well exposed to solvent and on the opposite side (Figure 7A).

## 3. Discussion

The purpose of this study was to identify and purify low molecular weight proteins from *Anemonia viridis* susceptible to have antiangiogenic activity not acting on the VEGF pathway. The purification protocol was set not to purify all compounds in *Anemonia viridis* having an antiangiogenic activity but only low molecular weight proteins. The reason is that synthetic proteins less than 50 residues can now be produced at low cost and have the great advantage to make possible a sterile production, which is not the case for monoclonal antibodies such as Avastin requiring a biological production. The high cost of Avastin is not related to the production as recombinant protein but to the purification process to have a germ free pharmaceutical production. A low molecular weight compound (<1000 Da) would be certainly less expensive to produce than a synthetic protein of 43 residues corresponding to BDS-5. However, a new chemical family of active principles requires now very expensive toxicological studies to have a Drug Master File suitable for clinical studies. Moreover, actual preclinical toxicity studies required for clinical studies are not sufficient to guarantee an absence of long term toxicities. These long-term toxicities are often due to accumulation in tissues of chemical compounds that cannot be or are insufficiently degraded. Therefore, a synthetic protein (with a molecular weight <5000 Da) represents a good compromise between cost of production and long-term safety issues.

This study shows that sea anemones are certainly a very interesting source of low molecular weight protein active principles, which have been understudied [1]. Although most of these proteins have three disulfide bridges and share sequence homologies, point mutations can totally change their structures and their pharmacological properties [1]. This is well outlined in this study if we compare BDS-5 with BDS-1. With its C-terminal alpha helix, BDS-5 appears to have a 3D structure different from sea anemone protein binding on ionic channels such as BDS-1. Proteins binding on ionic channel identified in sea anemones or in scorpions are characterized by a hydrophobic surface, which helps for a correct positioning of basic residues located on the opposite side. These basic residues create ionic bonds with acidic residues located in their ionic channel binding sites [6]. This is illustrated with BDS-1 that have a hydrophobic surface, with L18 and W35 (Figure 7B), and on the opposite side R12, which is one of the basic residues interacting with the binding site on potassium channel [7].

Although BDS-5 had never been purified and characterized, its sequence was previously known from a search for amino acid sequence motifs in sea anemone polypeptides, where 14 BDS-like sequences were identified in the *Anemonia viridis* genome [19]. This search was made in a cDNA library built from an Expressed Sequence Tag (EST) analysis performed on *Anemonia viridis* revealing 14,504 unique protein sequences in its genome [20]. Another EST analysis made it possible to discover BDS-15 [21]. Interestingly this study showed that BDS-1, BDS-3, BDS-4, BDS-5 and BDS-6 were the most represented transcripts among BDS like proteins [22]. The F18 mutation is found in half of the BDS like sequence. BDS-16 purified and sequenced in this study was never referenced before. Amazingly, the RGD motif is highly conserved in BDS sequences but no activity on integrins has been reported with BDS-1 or BDS-2. This is probably due to the N terminus in BDS-1 and BDS-2, which is masking a part of the RGD motif (Figure 7B).

Kunitz-Type Inhibitors (KTI) have a conserved scaffold (a chain of 40–60 residues stabilized by three disulfide bridges, a two-beta strand sheet and a short C terminal alpha helix) and the most known is the Bovine Pancreatic Trypsin Inhibitor [7]. Another property of KTI is to have an active site with amphoteric characteristic as described in the crystal structure of a Caribbean Sea anemone SHPI-1 in a complex with an Elastase enzyme used in this study [18]. The amphoteric characteristic is due to hydrophobic and hydrophilic poles made of F16 and R11 for SHPI-1 (Figure 7C), and F16 and R12 for BDS-5 (Figure 7A), respectively. A KTI from a tick was able to inhibit Trypsin, Elastase and interfere with blood vessel formation [22]. The RGD motif is recognized by Integrins that are membrane proteins playing a major role in cancer progression and metastasis [23]. BDS-5 appears to be a KTI with a RGD motif that could block angiogenesis in binding on integrins.

## 4. Materials and Methods

### 4.1. Phase Extractions and Filtrations from Crude Anemonia viridis

Living animals were collected in the south bay at Marseille in a harbor called “Base nautique du Prado” where shallow and calm water made the development of an important colony of at least 1000 *Anemonia viridis* possible. No other sea anemone species were observed in this spot. They are spread on little stones and they were collected alive fixed on their stone. The very same day the anemones (*n* = 10) with an average size of 10 cm were transported in sea water to the lab at the faculty of Pharmacy and the tentacles were immediately cut and frozen at −20 °C. Tentacles were then lyophilized (Jouan, Nante, France) for 3 days. The dried tentacles were mashed and suspended in tert-butyl-methyl-Ether (Merck, Lyon, France). After a first centrifugation at 4500 rpm for 20 min (Hettich, Vlotho, Germany), the organic liquid phase containing hydrophobic compounds was eliminated and the dry pellet was suspended in water containing 0.1% Trifluoroatic acid (TFA) (Carlo Erba, Barcelona, Spain). A second centrifugation at 4500 rpm for 20 min was carried out and the pellet was eliminated. The liquid phase containing hydrophilic compounds was then centrifuged in a 30 kDa Centrifugal Filter Unit (Millipore, Burlington, MA, USA) at 4500 rpm for 20 min and then lyophilized. The liquid extract containing compounds <30,000 Da was filtered at 0.22 µm with a filter (Millipore, Guyancourt, France) fixed on a syringe and then lyophilized. UV spectrum of the filtered solution was carried out from 240 to 340 nm with a Beckman DU640.

### 4.2. High Liquid Performance Chromatography (HPLC)

After filtration at 0.22 µm, the lyophilized material was suspended in water 0.1% TFA at 50 mg/mL. Purification was carried out using a Beckman HPLC System Gold 125 (Brea, CA, USA) apparatus with a Merck Lichrospher C8 reverse phase column (15 × 100 mm). Buffer A was water supplemented with 0.1% (*v*/*v*) TFA and buffer B was acetonitrile (Merck, Lyon, France) supplemented with 0.1% (*v*/*v*) TFA. Gradient was buffer B from 15% to 35% in 40 min, then 5 min at 90% B and a return to initial condition for 10 min at 15% B with a 2 mL/min flow rate. Fractions were collected manually and their absorptions were measured with a Beckman DU 640B before being frozen at −20 °C and lyophilized. HPLC analysis was carried out using a Merck Lichrospher RP-C8 column (4.6 × 100 mm) with similar buffers but a 0.8 mL/min flow rate using a linear gradient from 10% to 50% B in 40 min, then 50% to 90% B in 2 min, 5 min at 90% B, a return to initial condition in two min at 10% B and a final stationary phase of 10 min at 10% B. The total duration of the analytical run was 60 min. The UV absorbance of the effluent was measured from 220 to 330 nm with a diode ray Beckman 168 Gold detector and is expressed in milli Absorbance Unit (mAU).

### 4.3. VEGF Free HMEC Proliferation Assay

HMEC-1 cells were cultured in MCDB-131 medium containing 10% heat inactivated fetal bovine serum, 2 mmol/L glutamine, 100 units/mL penicillin and streptomycin, 1 mg/mL hydrocortisone and 10 ng/mL Epithelial Growth Factor. HMEC-1 cells were seeded on 0.1% gelatin-coated flasks and cultured at 37 °C with 5% CO_2_. HMEC-1 cells were transferred in 96-well plates at 5000 cells/well in culture medium. Twenty-four hours after seeding, cells were treated with extracts or the control vehicle solution. After 72 h of treatment, cells were exposed to 0.5 mg/mL of diMethyl Thiazol Tetrazolim (MTT) bromide for 3 h at 37 °C in culture medium. Cells were then washed with Phosphate Buffered Saline (PBS), formazan crystals were solubilized with 100 µL DMSO and absorbance was measured at 600 nm. MTT is degraded by a mitochondrial enzyme in living cells and forms a purple precipitate in mitochondria that is measured at 600 nm. 

### 4.4. Tubulogenesis Assays

HMEC-1 cells were cultured as described in the proliferation inhibition assay. HMEC-1 capillary network formation on Matrigel were performed on Matrigel™ (Gibco, Beziers, France) added to 96-well plates and allowed to solidify for 30 min at 37 °C. HMEC-1 cells (10,000 cells/well) were added atop the Matrigel™, treated with fractions or a control vehicle solution and incubated for 5 h at 37 °C. Capillary-like structures formed in the gel were photographed and their length was quantified using the METAMORPH 7.6 software [15].

### 4.5. Mass Determination and Sequencing

Matrix-Assisted Laser Desorption Ionization–Mass Spectrometry (MALDI-MS) spectra were obtained on an UltraflXtreme apparatus (Bruker, Bremen, Germany) operating in positive linear mode with delayed extraction. The samples were co-crystallized with a 10 mg/mL solution of Hydroxy α-Cyano-4-Cinnamic Acid (HCCA) on the MALDI-MS target by the dry droplet method. MALDI-MS spectra were acquired with an accelerating potential of 20 KV and a laser power set to the minimum level necessary to get an efficient signal. Spectrum mass calibration was based on external calibration using an appropriate peptides standard mixture (Peptide Calibration Standard, Brüker, Bremen, Germany). Sequencing was carried out with the Edman degradation method on a PPSQ31 B Shimadzu sequencer. Enzymatic digestion was carried out on 50 µg of the 28-min fraction and 1 µg a Trypsin/Lys C mixture (Promega, Madison, WI, USA) in ammonium bicarbonate 100 mM pH 9 at 37 °C. Different digestions were carried out from one hour to three days. For Nano-HPLC-MS/MS mass spectrometry, the 28-min fraction was reduced with DTT (Sigma-Aldrich, St. Louis, MO, USA), and alkylated with iodoacetamide (Sigma-Aldrich) and incubated overnight at 37 °C in a solution of 12.5 ng/μL of trypsin (Sequencing grade, Roche, Basel, Switzerland) in 25 mM NH4HCO3. The protein digests were sequenced by Nano-LC–MS/MS (Dionex RSLC coupled to a hybrid Q orbitrap mass spectrometer equipped with a Nano-ESI source; Q exactive ThermoFisher scientific, Waltham, WA, USA) in the data-dependent acquisition mode (method top 10). Data were matched to the Uniprot protein database. MALDI MS In Source Decay (ISD) was performed using an Ultraflextreme TOF/TOF mass spectrometer controlled by the FlexControl 3.3 software (BrukerDaltonics, Bremen Germany). The laser was increased (20%) to fragment protein in the source of the mass spectrometer using a specific matrix enabling the generation of hydrogen radicals breaking the peptide backbone producing C ions and z ions from 1000 to 5000 Da generating tag masses that could be used to search proteins in databases. Spectra were acquired in positive reflectron ion mode with 2000 laser shots accumulated and the laser power was set with an increase of 20% of fluensce with a frequency of 1000 Hz. The mass spectrometer parameters were set according manufacturer’s settings for optimal acquisition performance. Sequences were analyzed on Flex Analysis 3.0 software, (BrukerDaltonics, Bremen, Germany).

### 4.6. Far UV Absorption and Circular Dichroism (CD) Spectra

Spectra were measured with a 100 µm path length from 260 to 178 nm at 20 °C on a JASCO J-810 spectropolarimeter. Data were collected at 0.5 nm intervals using a step auto response procedure (JASCO, Oklahoma City, OK, USA) and the buffer background was subtracted. Protein concentrations were 1 mg/mL in 20 mM pH 7 phosphate buffer. Absorption spectra were measured to verify both concentration and light transmission. CD spectra are presented as ∆ε per amide rather than in ∆A unit to have a measure independent of mass and concentration according to the Beer Lambert law. Furthermore, the signal was divided by the number of amino acid residues of the protein minus one to have ∆ε per amide [17]. The instrument was calibrated with (+)-10-camphorsulfonic acid, a ratio of 2:1 was found between the positive CD band at 290.5 nm and the negative band at 192.5 nm.

### 4.7. Molecular Modelling

BDS-5 model was built with the Insight II software from MSI Technologies, Inc. (San Diego, CA, USA). The model was optimized with the Consistent Valence Force Field (CVFF) in term of the internal energies, using the van der Waals energy to monitor each step of the model. Model of BDS-5 was built with the InsightII Homology pulldown from the crystal structure of SHPI 1 in a complex with Elastase [19] available in the Brookhaven data bank (PDB code 1SHP). Minimization was performed with steepest descent and conjugate gradient algorithms. Dynamic was performed at 300 K for 1.1 ps using 1000 steps.

## 5. Conclusions

In conclusion, BDS-5 could be a member of a new family of antiangiogenic compounds, although the mechanism through which it acts has yet to be demonstrated. Further investigations are necessary to demonstrate that BDS-5 is acting without binding to VEGF or to the VEGF receptor. BDS-5 appears to be structurally related to Kunitz-type inhibitor and its antiangiogenic activity might be due to an interaction with an Integrin due to a RGD motif well exposed to the solvent. BDS-5 could improve cancer treatment in complementing existing antiangiogenic compounds binding on the VEGF in antiangiogenic combinatory therapies. Complementary pharmacological characterization and structural study with 2D NMR are planned with BDS-5. Chemical synthesis of BDS-5 might open the road to a clinical development in cancer therapy, particularly for children, for whom anti-mitotic compounds have devastating side effects.

## Supplementary Materials

The following are available online at http://www.mdpi.com/1660-3397/16/4/134/s1, Figure S1: HMEC tubulogenesis assay with the 28’ fraction with concentrations going from 10 µM to 3.2 nM in a triplicate experiment. A network with interconnected lines similar to control was observed only at 3.2 nM. Figure S2: High resolution nano-HPLC MS of fraction 28’. The numbers in square indicate the charge state of the peptides, the insert shows the isotopic envelopes of the quintupled charged main species. Figure S3: Maldi ISD of fraction 28. A: annotation of the ions obtained by Maldi ISD of the three main species of the fraction. B: The insert shows the common n-terminal part of BDS1 and BDS5. Figure S4: MALDI spectra of digest of the 28’ fraction after reduction before Nano-HPLC ms/ms analysis on Orbitrap Q-exactive: The peaks 1 to 4 match with the sequence of BDS-5 (table in insert), A is specific to BDS1 with a Leu/PHE mutation, B match with the BDS16 sequence and was de Novo sequenced by hplc-ms/ms as shown below. Figure S5: MS/MS spectrum of the peptide of the N-terminal part of BDS-16 with two mutations (S5 et P7 instead of F5 et S7 for BDS1 and BDS5). The spectrum is annotated by the y-ions generated by the HCD fragmentation from the Q-exactive Orbitrap. Figure S6: Digestion of the non-reduced fraction from 30 to 70 h. A: 30 H, B: 40 H C: 70 H, D: Reduction of the digest 70 H.
