# Supplementary material for: A Low Molecular Weight Protein from the Sea Anemone Anemonia viridis with an Anti-Angiogenic Activity
Source: Mar Drugs. 2018 Apr 19;16(4):134. doi: 10.3390/md16040134 (PMC5923421; doi:10.3390/md16040134)
Supplement: Supplementary file 1 [file marinedrugs-16-00134-s001.pdf]

A low molecular weight protein from the Sea Anemone *Anemonia viridis* with an anti Angiogenic activity.

Supplementary results

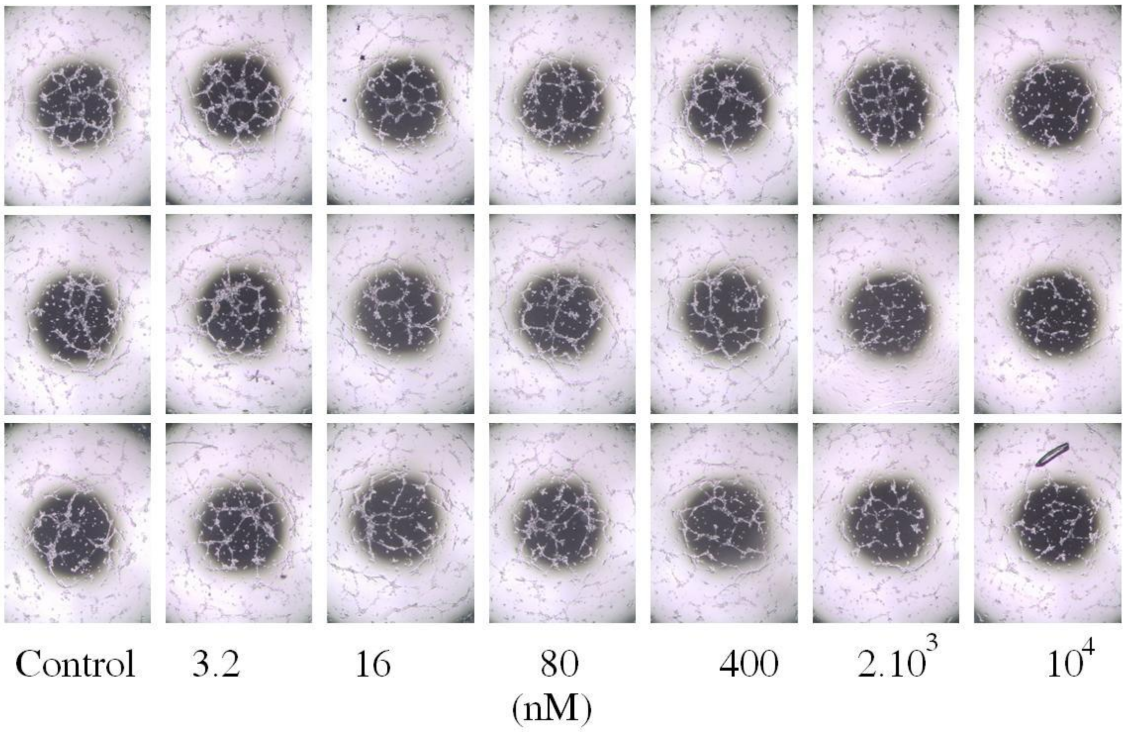

Figure 1S. HMEC tubulogenesis assay with the 28' fraction with concentrations going from 10 µM to 3.2 nM in a triplicate experiment. A network with interconnected lines similar to control was observed only at 3.2 nM.

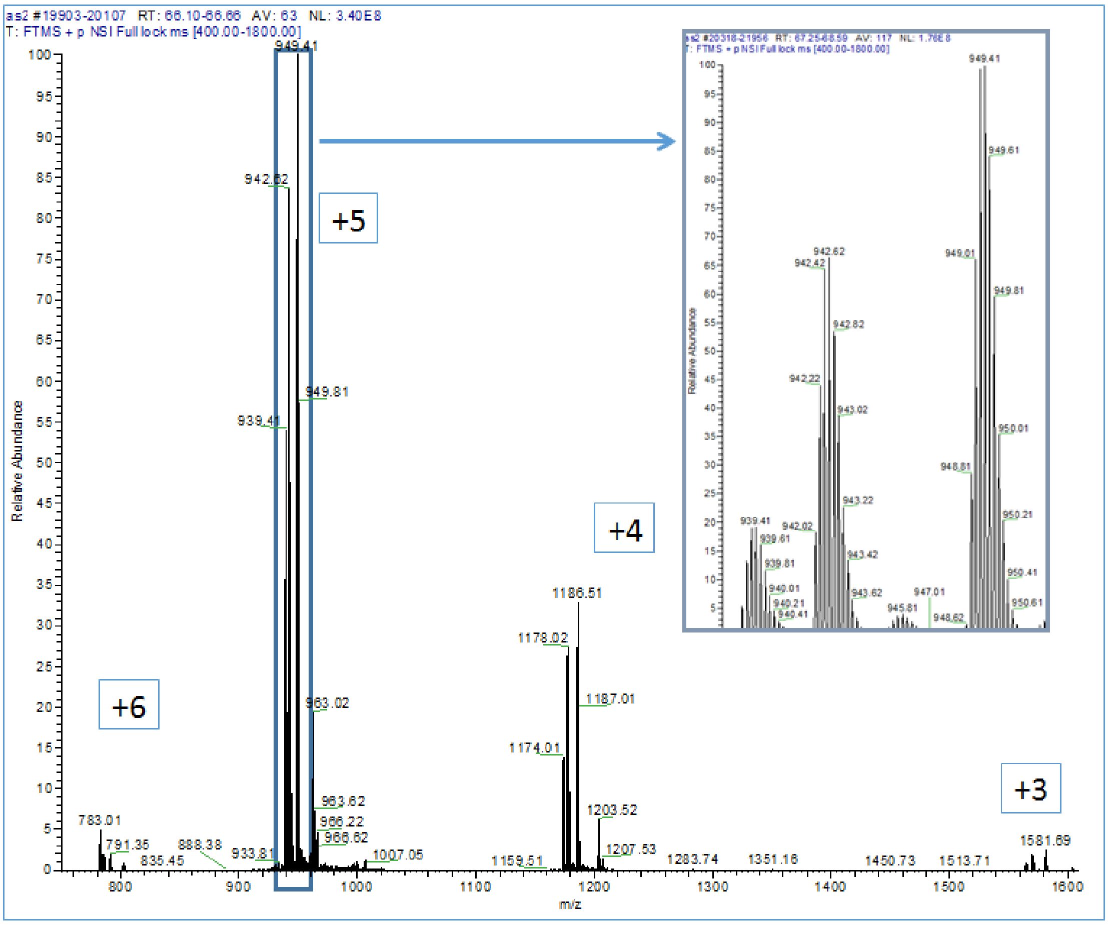

Fig 2S. High resolution nano-HPLC MS of fraction 28'. The numbers in square indicate the charge state of the peptides, the insert shows the isotopic envelopes of the quintupled charged main species

.

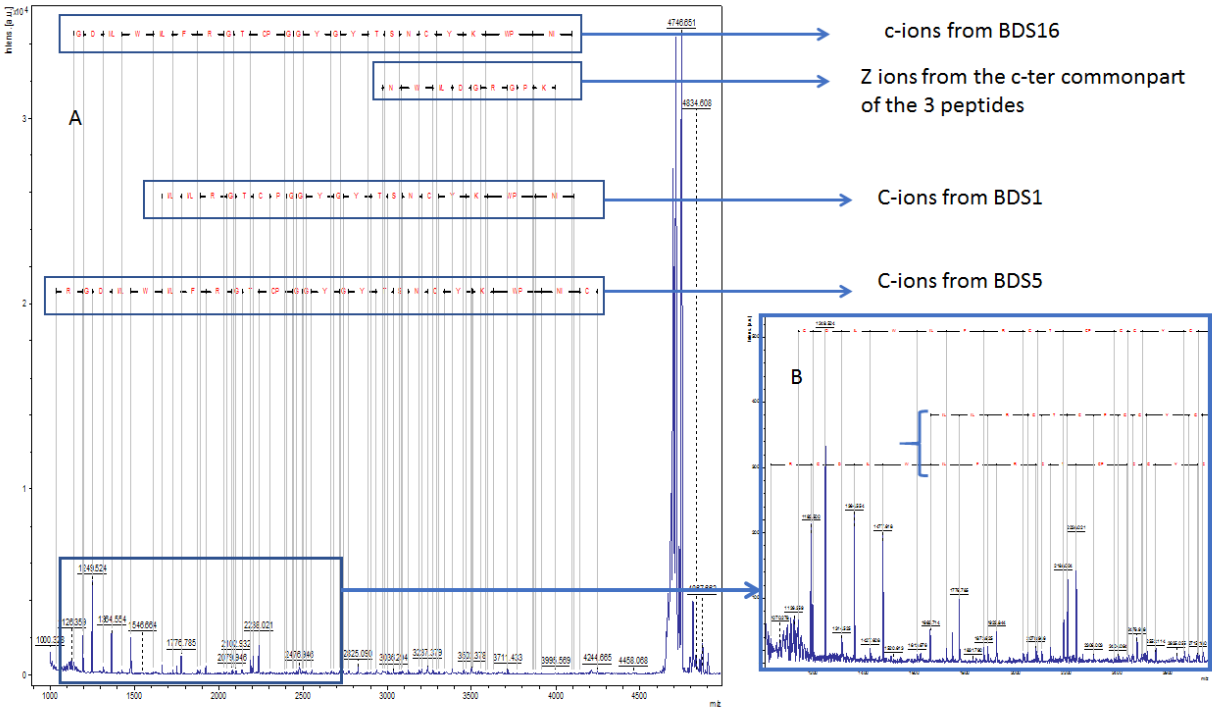

Fig 3S. Maldi ISD of fraction 28. A:annotation of the ions obtained by Maldi ISD of the three main species of the fraction. B: The insert shows the common n-terminal part of BDS1 and BDS5.

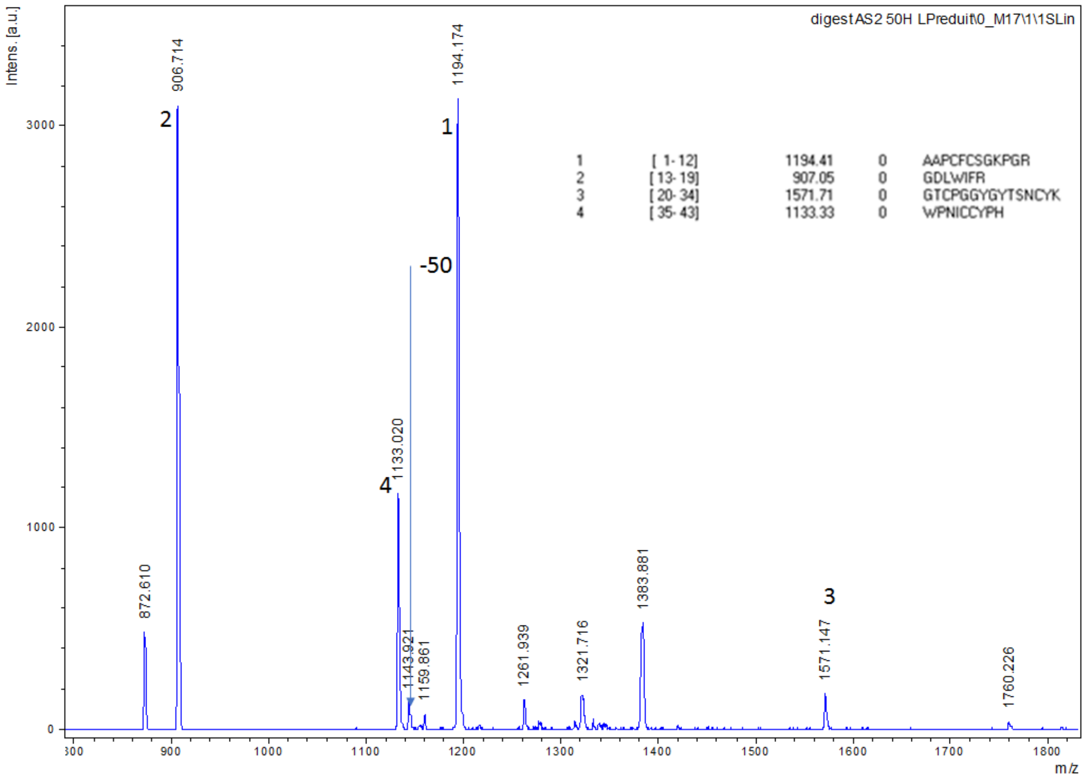

Fig 4S. MALDI spectra of digest of the 28' fraction after reduction before Nano-HPLC ms/ms analysis on Orbitrap Q-exactive: The peaks 1 to 4 match with the sequence of BDS-5(table in insert), A is specific to BDS1 with a Leu/PHE mutation, B match with the BDS16 sequence and was de Novo sequenced by hplc-ms/ms as shown below.

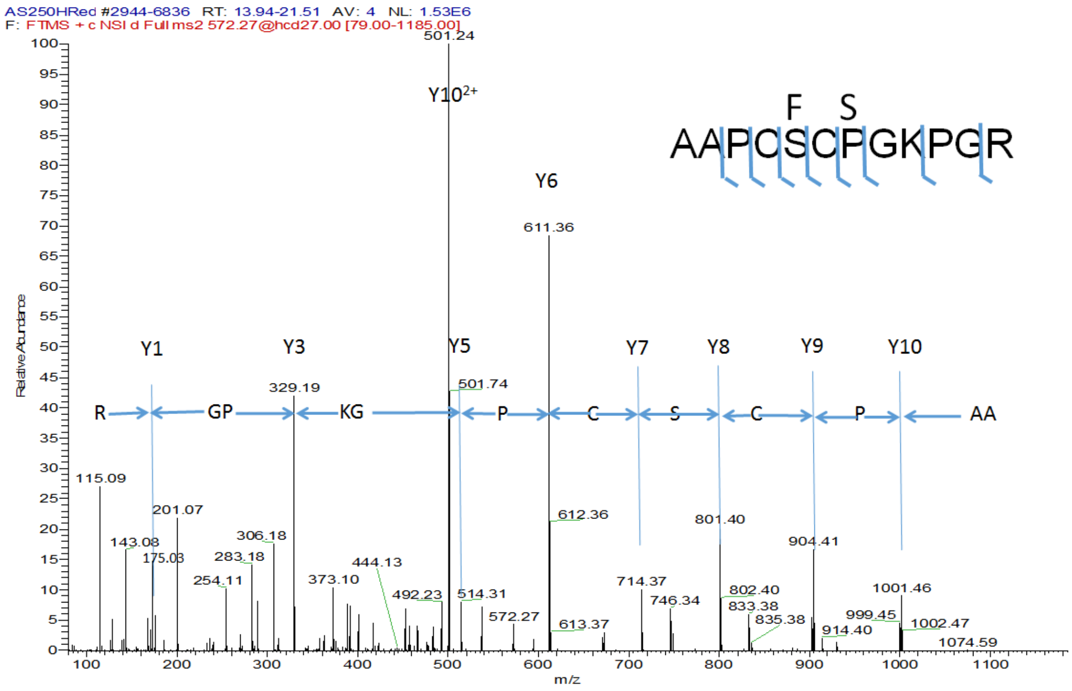

Fig 5S.MS/MS spectrum of the peptide of the N-terminal part of BDS-16 with two mutations (S5et P7 instead of F5 et S7 for BDS1 and BDS5). The spectrum is annotated by the y-ions generated by the HCD fragmentation from the Q-exactive Orbitrap.

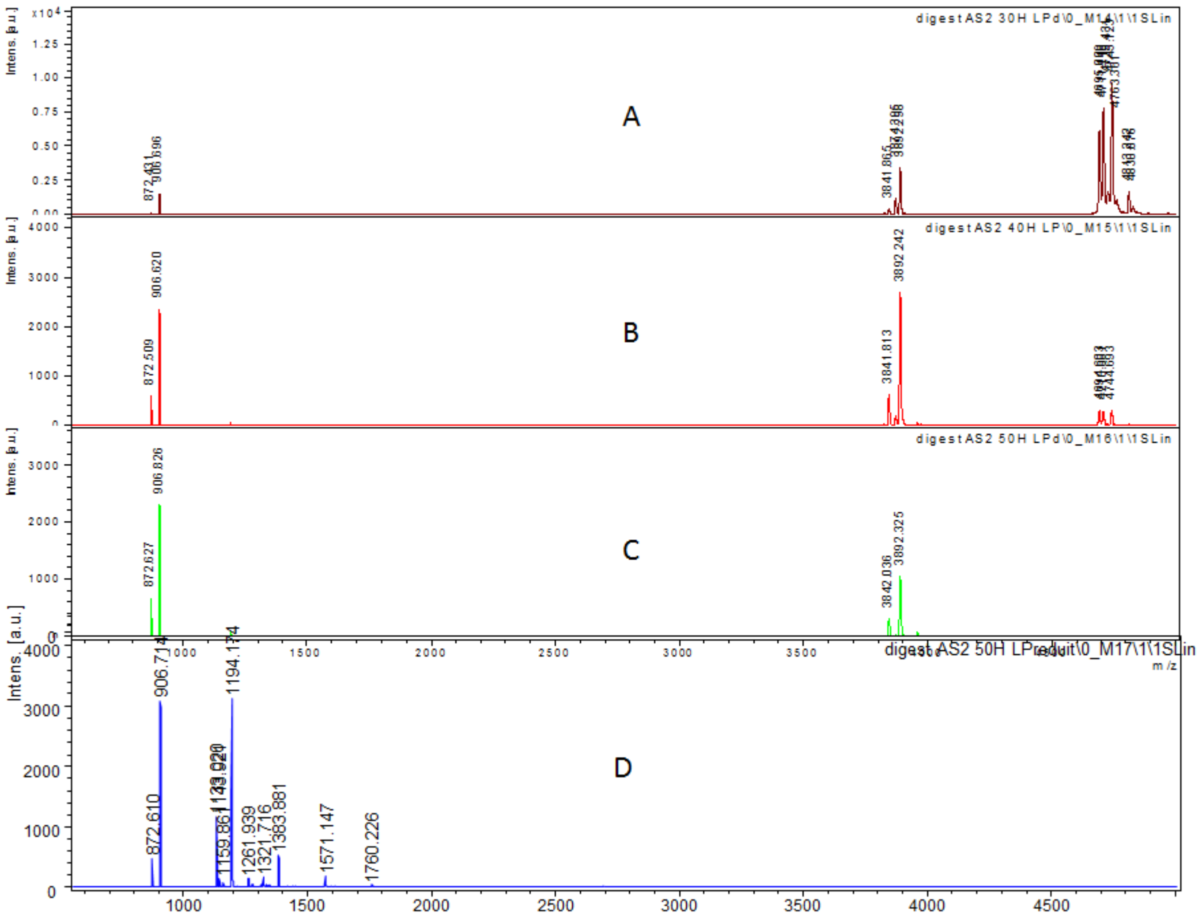

Fig 6S.Digestion of the non-reduced fraction from 30 to 70 hours. A: 30 H, B: 40 H C: 70 H
D: Reduction of the digest 70H
